# LETM1 (leucine zipper-EF-hand-containing transmembrane protein 1) silence reduces the proliferation, invasion, migration and angiogenesis in esophageal squamous cell carcinoma via KIF14 (kinesin family member 14)

**DOI:** 10.1080/21655979.2021.1982275

**Published:** 2021-10-04

**Authors:** Qiang Zhao, Sheng Chen, Lu Chen

**Affiliations:** a Cancer Hospital of the University of Chinese Academy of Sciences (Zhejiang Cancer Hospital), Institute of Basic Medicine and Cancer (Ibmc), Chinese Academy of Sciences, Zhejiang, Hangzhou, P.R. China; b Department of Surgical Oncology, Cancer Hospital of the University of Chinese Academy of Sciences (Zhejiang Cancer Hospital), Institute of Basic Medicine and Cancer (Ibmc), Chinese Academy of Sciences, Hangzhou, Zhejiang, P.R. China

**Keywords:** LETM1 (leucine zipper-EF-hand-containing transmembrane protein 1), KIF14 (kinesin family member 14), esophageal squamous cell carcinoma (ESCC), angiogenesis

## Abstract

Esophageal squamous cell carcinoma (ESCC), a major form of esophageal cancer, is a serious threat to human health. This study was conducted to investigate the pathogenesis of ESCC and find effective therapies to improve it. Protein expression of transfected plasmids was detected by RT-qPCR and western blot. Co-immunoprecipitation assay was performed to verify the binding of LETM1 and KIF14. CCK-8, wound healing and transwell assays were used to assess the proliferation, invasion and migration of ESCC cells. Finally, the angiogenesis was assessed using tubule formation assay. The co-immunoprecipitation results showed that LETM1 could bind to KIF14. The cytological and protein results demonstrated that interference with LETM1 caused downregulation of KIF14 expression, which led to inhibition of proliferation, invasion, migration and angiogenesis in ESCC cells. Taken together, interfering with LETM1 to downregulate KIF14 may become a new target for ESCC treatment.

## Introduction

As one of the most common malignant tumors worldwide, esophageal cancer seriously threatens human health [1–3]. Currently, the pathogenesis of esophageal squamous cell carcinoma (ESCC), a major form of esophageal cancer, has not been fully understood yet. Meanwhile, there are extremely few markers available for ESCC diagnosis and treatment. Hence, it is of great need to better understand the pathogenic processes of ESCC.

Leucine zipper-EF-hand-containing transmembrane protein 1 (LETM1), a mitochondrial endosomal protein, is highly expressed in many human malignancies, such as gastric adenocarcinoma [4], non-small cell lung carcinoma [5] and colorectal adenocarcinoma [6], and has a close relationship with poor prognosis. Remarkably, it has been found that LETM1 expression was increased in ESCC tissues and the higher the LETM1 expression, the worse the prognosis [7]. However, the role and mechanism of LETM1 involved in the biological activity of esophageal squamous carcinoma have not been well-studied. Thus, the present study first demonstrated the effects of LETM1 interference on ESCC through a series of experiments. In addition, to further explore the possible mechanism of action of LETM1, related genes bound by LETM1 were searched through Biogrid database (https://thebiogrid.org/).

As a member of LETM1-binding genes, kinesin family member 14 (KIF14), a potential oncogene, exhibited promotive effects on the malignant development of many cancers, including gastric cancer [8], colorectal cancer [9] and glioma [10]. Previous studies have verified that KIF14 was overexpressed in ESCC [11,12]. Therefore, it is meaningful to investigate LETM1 and KIF14 together.

Therefore, this study aims to investigate the pathogenesis of esophageal squamous carcinoma and find effective therapeutic approaches by investigating the relationship between the roles of LETM1 and KIF14 in ESCC cells.

## Materials and methods

### Cell culture and transfection

Human esophageal epithelial cell line (Het-1A) and human ESCC cell lines (KYSE150, TE10, HCE7, KYSE450 and TE11) were purchased from the Cell Bank of the Chinese Academy of Sciences. Cells were cultured in Dulbecco's modified eagle medium (DMEM; Gibco) containing 10% fetal bovine serum (FBS) with 5% CO_2_ at 37°C and passaged on alternate days. The cell lines with the highest expression of LETM1 or KIF14 were used for the subsequent experiments.

For transfection, small interfering RNA (siRNA) targeting LETM1 (siRNA-LETM1-1 and siRNA-LETM1-2), corresponding negative control (siRNA-NC), KIF14 overexpression plasmids (Ov-KIF14) and pcDNA3.1 empty vector (Ov-NC) were obtained from Shanghai GenePharma company and transfected into TE11 cells by Lipofectamine™ 2000 transfection reagent (Thermo Fisher Scientific, Inc.) according to the manufacturer’s instruction.

### Reverse transcription-quantitative polymerase chain reaction (RT-qPCR)

Total RNA was extracted from cell lysates using TRIzol® reagent. With the adoption of PrimeScript™ RT reagent kit (Takara Bio, Inc.), the extracted RNA was reverse-transcribed into complementary DNA (cDNA). Subsequently, qPCR reaction was performed on an ABI 7500 quantitative PCR instrument (Applied Biosystems, CA, USA) with SYBR Premix Ex Taq reagents (Takara, Tokyo, Japan). The PCR condition was 95°C for 30 s followed by 40 cycles of amplification (95°C for 5 s and 60°C for 30 s). GAPDH was used as an internal control, and the relative gene expression was determined by the 2^−∆∆Ct^ method [13]. The primer pairs used are given in Table 1.

**Table 1.** List of specific primer pairs for target and reference genes for RT-qPCR analysis

Gene	Primer name	Sequence 5ʹ-3’
LETM1	Forward	CCGAGTGCCTTCGCATAGTG
	Reverse	ACTTCTCTACTACCGAGTCATCG
KIF14	Forward	CCTCACCCACAGTAGCCGA
	Reverse	AAGTGCCAATCTACCTACAGGA
U6	Forward	AAAGCAAATCATCGGACGACC
	Reverse	GTACAACACATTGTTTCCTCGGA

### Western blotting assay

The total proteins of ESCC cells were extracted using RIPA lysis buffer, and their concentration was quantified using bicinchoninic acid (BCA) kits (Thermo Fisher Scientific Inc.). The proteins were separated by SDS-PAGE and then transferred to PVDF membranes. After blocking with 5% skim milk, PVDF membranes were incubated with the primary antibody at 4°C overnight and subsequently incubated with secondary antibody for another 2 h. Finally, the protein signal was detected by adding enhanced chemiluminescence reagents, and the Image-Pro Plus version 6.0 software (Roper Technologies, Inc.) was used to quantify protein expression levels.

### Cell proliferation assay

Cell proliferation ability was assayed using CCK-8 assay. Briefly, the transfected cells were inoculated into 96-well plates and incubated at 37°C with 5% CO2. Subsequently, the cells were cultured for 24, 48, and 72 hours, respectively, and then incubated with CCK-8 reaction solution at 37°C for 30 min. Finally, optical density values were detected at 450 nm using an enzyme marker and cell proliferation curves were plotted.

### Co-immunoprecipitation (CoIP) assay

According to Biogrid database, LETM1 can be bound to KIF14. To further verify this result, Co-IP assay was performed. Proteins were extracted as described in western blot assay. Protein samples were incubated with 50% protein A/G agarose bead solution for 2 h at 4°C. Subsequently, IgG and LETM1 or KIF14 antibodies were added and shaken slightly at 4°C overnight. The sample was centrifuged at 1000 x g for 5 min, and the precipitation was washed with Co-IP buffer three times. Finally, the obtained proteins were analyzed by western blot.

### Transwell assay

To check the invasive ability of TE11 cells, Transwell assay was employed. Cells were seeded in the upper part of the Transwell chamber with 8 μm wells pre-coated with Matrigel and cultured in RPMI-1640 medium. Afterward, the medium containing 5% FBS was incorporated into the lower chamber as a chelating agent. Cells were cultured for 24 hours, fixed with 4% paraformaldehyde, and stained with 0.1% crystal violet at 37°C. Images were taken with an inverted light microscope (magnification, x100), and the number of invading cells in each well was counted from a randomly selected area.

### Wound healing assay

Cells were cultured in 6-well culture plates. After reaching 80–90% confluence, adherent cells were scratched with a 10 µl pipette tip and starved. Thereafter, the scratched area was pictured with a light microscope at 0 h and 24 h (magnification, x100). The images were further quantified by ImageJ software.

### Tube formation assay

Human umbilical vein endothelial cells (HUVECs) were inoculated into 96-well plates containing Matrigel® gel substrates and incubated with transfected cell supernatants for 8 h at 37°C with 5% CO_2_. HUVEC cells in five random fields were observed using a light microscope (magnification, x100). Statistics were performed using ImageJ software.

### Statistical analysis

Data were expressed as the mean ± standard deviation (SD) of at least three independent experiments. Values between group means were compared by one-way ANOVA and Tukey’s test. Standard error bars were presented for all data points. Statistical analysis was performed using GraphPad Prism 8.0 software (GraphPad Software, Inc.). p < 0.05 was considered to be a significant deviation.

## Results

### The expression of LETM1 was upregulated in ESCC cells

**Figure 1.**
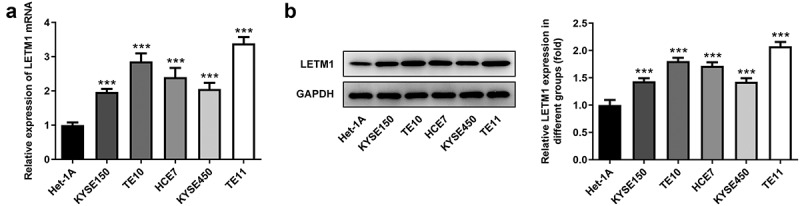
The expression of LETM1 in ESCC cells. (a) The mRNA expression of LETM1 in ESCC cells; (b) The protein expression of LETM1 in ESCC cells. ***P < 0.001 vs. Het-1A

The mRNA and protein levels of LETM1 in ESCC cells were detected using RT-qPCR and western blot, respectively. The results showed that LETM1 was significantly expressed in ESCC cells compared to Het-1A cells (Figures 1A-1B). Among them, notably, mRNA expression of LETM1 in TE11 cells was 3.4-fold higher than that in Het-1A cells and LETM1 protein expression in TE11 cells was 2.1-fold higher than that in Het-1A cells. Therefore, TE11 was selected as a model cell line for the subsequent experiments.

### *LETM1 silence inhibited cell proliferation, invasion, migration and* angiogenesis of ESCC

**Figure 2.**
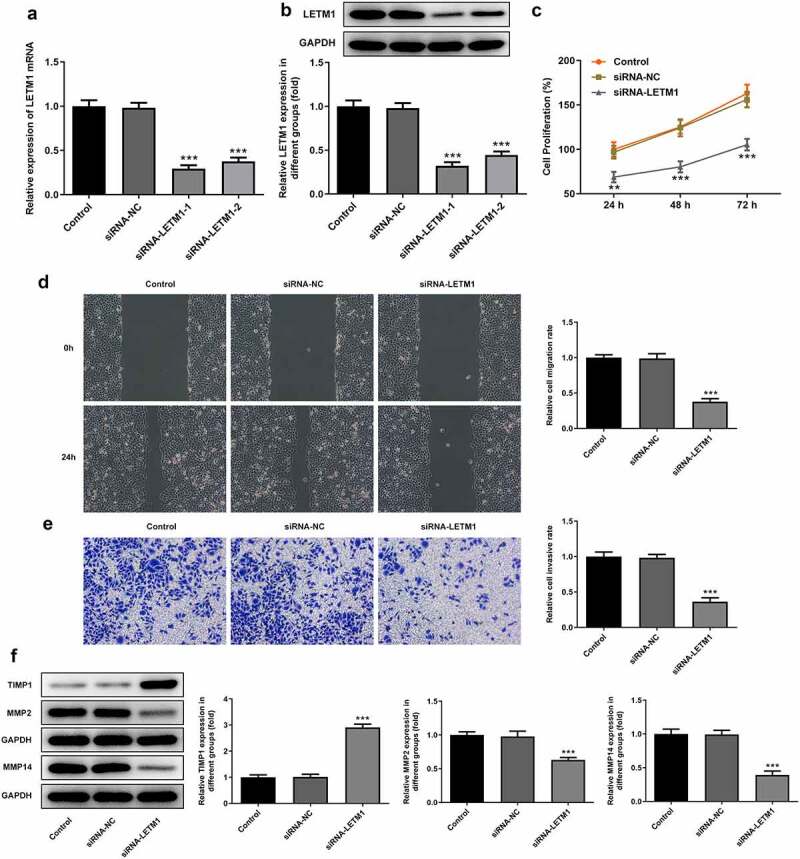
The results of cell proliferation, invasion and migration in ESCC. (a) The mRNA expression of LETM1 in transfected cells; (b) The protein expression of LETM1 in transfected cells; (c) The result of ESCC cell proliferation; (d) The result of wound healing assay in ESCC cells; (e) The result of transwell assay in ESCC cells; (f) The results of cell invasion and migration-related protein expression. **P < 0.01, ***P < 0.001 vs. siRNA-NC

**Figure 3.**
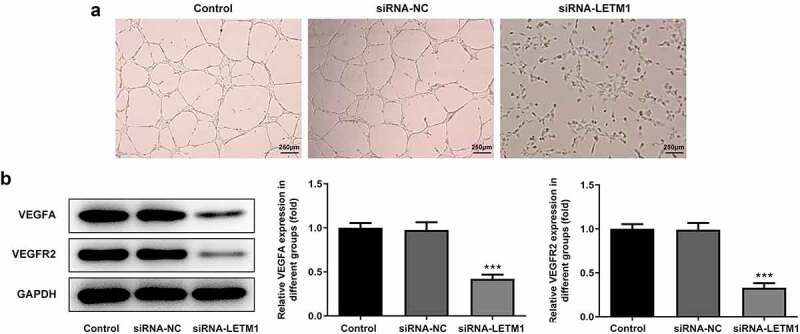
The results of angiogenesis in ESCC. (a) The pictures of tube formation; (b) The related protein expression of tube formation. ***P < 0.001 vs. siRNA-NC

To explore the effects of LETM1 silence on proliferation, invasion and migration of ESCC, TE11 cells were transfected with siRNA-LETM1 plasmids. The results showed that the silencing effect of siRNA-LETM1-1 was better comparable to that of siRNA-LETM1-2, so siRNA-LETM1-1 was used as LETM1-silenced cells (siRNA-LETM1 group) for the next study (Figures 2A-2B). Besides, the results indicated that the proliferation of ESCC cells was significantly inhibited by LETM1 knockdown compared with the siRNA-NC group (Figure 2c). Meanwhile, the results of wound healing and Transwell assay showed that the cell invasion and migration were remarkably suppressed in siRNA-LETM1 group compared with the siRNA-NC group (Figures 2D-2E). The upregulation of TIMP1 and the downregulation of MMP2 and MMP14 were detected by western blotting assay (figure 2f), which further validated that interfering LETM1 could inhibit cell invasion and migration. In addition, the results of tube formation indicated that interfering with LETM1 had an oppressive effect on HUVEC (Figure 3a). Knockdown of LETM1 significantly reduced the protein levels of VEGFA and VEGFR2 (Figure 3b). So, the results demonstrated that LETM1 silence could inhibit angiogenesis of ESCC.

### LETM1 could bind to KIF14 in ESCC cells

**Figure 4.**
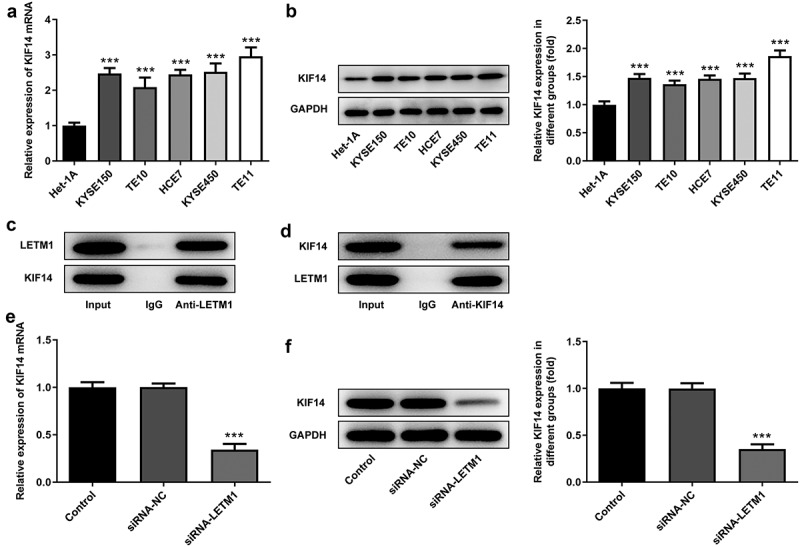
LETM1 in conjunction with KIF14 in ESCC cells. The (a) mRNA and (b) protein expression of KIF14 in ESCC cells, ***P < 0.001 vs. Het-1A; (c-d) The results of CoIP assay; The (e) mRNA and (f) protein expression of KIF14 in transfected cells, ***P < 0.001 vs. siRNA-NC

The mRNA and protein expression of KIF14 in different cell lines was detected using RT-qPCR and western blot, respectively. Results showed that KIF14 was highly expressed in human ESCC cell lines (Figures 4A-4B). Moreover, it is noteworthy that the mRNA expression of KIF14 in ESCC TE11 cells was 2.9-fold higher than that in normal esophageal epithelial cells and the protein expression was 1.9-fold higher. Therefore, the ESCC TE11 cell line was chosen to be performed in the follow-up experiments. The results of CoIP assay showed that protein LETM1 and protein KIF14 could be precipitated by each other, further supporting the existence of LETM1 and KIF14 interactions (Figures 4C-4D). Subsequently, the expression of KIF14 in siRNA-LETM1 transfected cells was detected by RT-qPCR and western blot. What is more, it was also found that the mRNA and protein expression of KIF14 was significantly reduced in siRNA-LETM1 transfected cells (Figures 4E-4F), suggesting that knockdown of LETM1 was accompanied by downregulation of KIF14 expression.

### *LETM1 silence inhibited cell proliferation, invasion, migration and* angiogenesis *of ESCC via targeting KIF14.*

**Figure 5.**
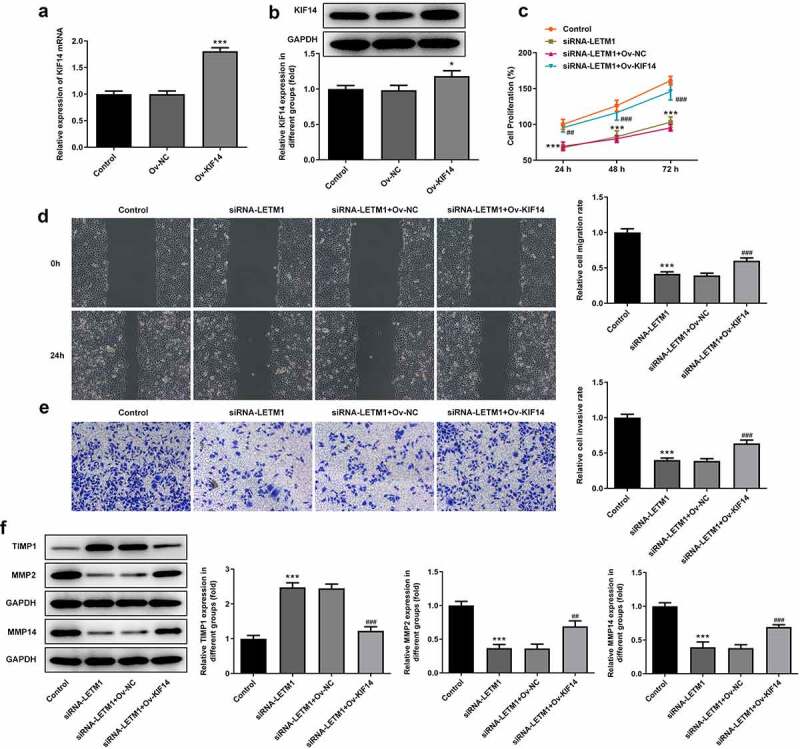
The results of cell proliferation, invasion and migration in ESCC affected by LETM1 and KIF14. The (a) mRNA and (b) protein expression of KIF14 in transfected cells, *P < 0.05, ***P < 0.001 vs. Ov-NC; (c) The result of ESCC cell proliferation; (d) The result of wound healing assay in ESCC cells; (e) The result of transwell assay in ESCC cells; (f) The results of cell invasion and migration-related protein expression. ***P < 0.001 vs. control; ^##^P < 0.01, ^###^P < 0.001 vs. siRNA-LETM1+ Ov-NC

KIF14 expression gained a huge growth after transfection with KIF14 overexpression plasmids (Figures 5A-5B). Obviously, the decreased proliferation of TE11 cells caused by LETM1 silence was then revived by KIF14 overexpression in comparison with siRNA-LETM1+ Ov-NC (Figure 5c). However, when KIF14 was overexpressed, cell proliferation was promoted compared to LETM1 silencing group; compared with the control group, no significant change in the proliferation was observed in LETM1 silencing added Ov-KIF14 group (siRNA-LETM1+ Ov-KIF14). It can be seen that interfering LETM1 inhibited ESCC proliferation via KIF14. Compared with the control group, cell invasion and migration in siRNA-LETM1 group were considerably inhibited (Figures 5D-5E). But with KIF14 overexpression, cell invasion and migration were suppressed to a lesser extent. At the same time, compared with the siRNA-LETM1+ Ov-NC group, the inhibition of siRNA-LETM1+ Ov-KIF14 group was significantly reduced on cell invasion and migration. In addition, the results of protein assay showed that the protein expression of MMP2 and MMP14 was significantly upregulated and TIMP1 expression was significantly downregulated in siRNA-LETM1+ Ov-KIF14 group compared to siRNA-LETM1+ Ov-NC group (figure 5f).

**Figure 6.**
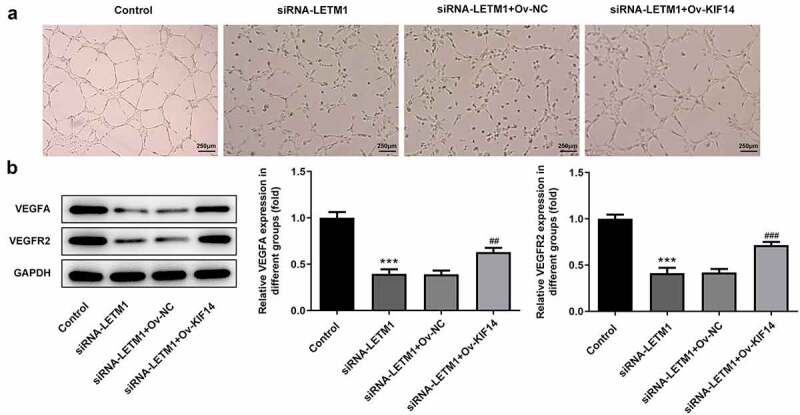
The results of angiogenesis in ESCC effected by LETM1 and KIF14. (a) The pictures of tube formation; (b) The related protein expression of tube formation. ***P < 0.001 vs. control; ^##^P < 0.01, ^###^P < 0.001 vs. siRNA-LETM1+ Ov-NC

Notably, the results of tube formation demonstrated that KIF14 overexpression reversed the inhibitory effect of LETM1 silencing on HUVEC, evidenced by the increased angiogenic ability of TE11 cells (Figure 6a). Moreover, the decreased expressions of VEGFA and VEGFR2 induced by LETM1 silence were partially reversed after transfection with KIF14 overexpression plasmids, implying that LETM1 silence exhibited inhibitory effects on VEGFA and VEGFR2 expressions through regulating KIF14 (Figure 6b).

## Discussion

Esophageal cancer is a gastrointestinal malignancy with high aggressiveness [14]. ESCC is the most commonly observed sub-type of esophageal cancer, accounting for approximately 70–90% of esophageal cancer cases [15–17]. It is worth noting that metastasis is the leading cause of ESCC death [17–19]. However, the metastasis process is complex, including the growth, transformation, invasion, migration and angiogenesis of tumor cells as well as the alteration of the tumor microenvironment [20,21]. More importantly, during esophageal cancer progress, the overexpression of pro-angiogenic factors promotes pathological angiogenesis, resulting in a local unbalance between pro-angiogenic and anti-angiogenic factors [22]. Angiogenesis, as one of the cancer markers, needs to be investigated in ESCC studies. Thus, in this study, we first hypothesized and demonstrated that LETM1 silence down-regulated KIF14 to inhibit proliferation, invasion, migration and angiogenesis of ESCC cells via regulating KIF14 expression.

As a mitochondrial inner membrane protein, LETM1 has been a potential target marker for a variety of cancer studies. It has been reported that high expression of LETM1 reduced the survival rate of gastric cancer patients [4]. In addition, LETM1 elevated the expression of proteins involved in signaling pathways, such as PI3K/Akt signaling pathway. In the previous studies, it is interesting to note that the analysis of gastric cancer tissue sections and gastric cancer cells revealed that knockdown of LETM1 greatly inhibited the cancer cell growth, migration and invasion and affected cell distribution [4,23]. Meanwhile, high expression of LETM1 in renal cell carcinoma has also been reported [24]. Interfering with LETM1 downregulated related proteins and significantly reduced cancer cell proliferation, migration and invasion via a downstream control of Wnt/β-Catenin signaling pathway.

Notably, Yang et al. investigated the expression of LETM1 in ESCC using a number of clinical tissue specimens. Their findings demonstrated that the expression of LETM1 was significantly elevated in ESCC; thus, LETM1 may be a new target for ESCC therapy [7]. However, their study did not pay much attention to the role of LETM1 pathway in regulating ESCC. Thus, in this research, the mRNA and protein expression levels of LETM1 in ESCC cells were examined first and the results showed that the expression of LETM1 in ESCC cells was remarkably enhanced compared with that in esophageal epithelial cells, which was consistent with the results of tissue samples in the study by Yang [7]. With more studies, we were gratified to find that knockdown of LETM1 significantly reduced ESCC cell proliferation, invasion and migration, which was consistent with the role of LETM1 in other cancers. More excitingly, LETM1 silence had a good inhibited effect on angiogenesis in ESCC.

To further explore the mechanism of the role of LETM1 in ESCC, we searched for unique interactors that could bind to LETM1 by using the Biogrid database. Fortunately, KIF14 was found to be an interactor that could combine with LETM1. It is important to note that KIF14 has been reported by several researchers as a potential factor for carcinogenesis. In terms of glioma, previous studies have shown that overexpression of KIF14 could promote glioma cell migration, invasion and angiogenesis [10]. Meanwhile, tumor cell growth was inhibited and apoptosis was promoted by silencing KIF14 in glioblastoma [25]. In addition, some researchers found that KIF14 may be closely related to tumorigenesis in alimentary tract cancers [26]. Through the analysis of blood and tissue samples, it was demonstrated that high expression of KIF14 was involved in tumorigenesis. For example, the expression of KIF14 was remarkably increased in gastric cancer tissues and cells. And a higher level of KIF14 expression was shown to be associated with a worse prognosis. Moreover, KIF14 silence led to diminished proliferation, invasion and migration of gastric cancer cells [8,27]. There are also some noteworthy examples of ESCC. The investigators reported that KIF14 was overexpressed in ESCC clinical specimens [11]. Additionally, it was also found that the reduction in KIF14 expression might significantly inhibit the proliferation of ESCC cells and enhance the sensitivity of tumors to chemotherapy [28].

In this study, it was successfully verified for the first time that LETM1 and KIF14 could bind to each other. Meanwhile, the expression of KIF14 was greatly reduced after knocking down LETM1, demonstrating that KIF14 might be a downstream factor of LETM1. To further accurately demonstrate the relationship between LETM1 and KIF14, a sequence of experiments were performed on ESCC cells with LETM1 knockdown and/or KIF14 overexpression. As an exciting result, the overexpression of KIF14 significantly reversed the inhibited effect on cancer cells induced by LETM1 silence.

## Conclusion

The expressions of LETM1 and KIF14 were detected in ESCC cells and their relationship was clarified by disrupting LETM1 and/or upregulating KIF14. To our knowledge, this is the first report proving that LETM1 regulates tumor cell behavior via targeting KIF14. Therefore, knocking down LETM1/KIF14 may contribute to the study of molecular mechanisms of ESCC.

## Highlights

(1) LETM1 and KIF14 were significantly expressed in ESCC cells.

(2) LETM1 and KIF14 could bind to each other.

(3) KIF14 was greatly reduced after knocking down LETM1.

(4) KIF14 significantly reversed the inhibited effect on cancer cells induced by LETM1 silence.
